# Analysis of Brain MRI Images Using Improved CornerNet Approach

**DOI:** 10.3390/diagnostics11101856

**Published:** 2021-10-08

**Authors:** Marriam Nawaz, Tahira Nazir, Momina Masood, Awais Mehmood, Rabbia Mahum, Muhammad Attique Khan, Seifedine Kadry, Orawit Thinnukool

**Affiliations:** 1 Department of Computer Science, University of Engineering and Technology, Taxila 47050, Pakistan; marriam.nawaz@uettaxila.edu.pk (M.N.); Tahira.nazir77@gmail.com (T.N.); momina.masood@uettaxila.edu.pk (M.M.); awias.mehmood@uettaxila.edu.pk (A.M.); rabbia.mahum@uettaxila.edu.pk (R.M.); 2 Department of Computer Science, HITEC University Taxila, Taxila 47080, Pakistan; attique@ciitwah.edu.pk; 3 Department of Applied Data Science, Noroff University College, 4612 Kristiansand, Norway; seifedine.kadry@noroff.no; 4 Research Group of Embedded Systems and Mobile Application in Health Science, College of Arts, Media and Technology, Chiang Mai University, Chiang Mai 50200, Thailand

**Keywords:** deep learning, medical imaging, MRI, CornerNet, DenseNet

## Abstract

The brain tumor is a deadly disease that is caused by the abnormal growth of brain cells, which affects the human blood cells and nerves. Timely and precise detection of brain tumors is an important task to avoid complex and painful treatment procedures, as it can assist doctors in surgical planning. Manual brain tumor detection is a time-consuming activity and highly dependent on the availability of area experts. Therefore, it is a need of the hour to design accurate automated systems for the detection and classification of various types of brain tumors. However, the exact localization and categorization of brain tumors is a challenging job due to extensive variations in their size, position, and structure. To deal with the challenges, we have presented a novel approach, namely, DenseNet-41-based CornerNet framework. The proposed solution comprises three steps. Initially, we develop annotations to locate the exact region of interest. In the second step, a custom CornerNet with DenseNet-41 as a base network is introduced to extract the deep features from the suspected samples. In the last step, the one-stage detector CornerNet is employed to locate and classify several brain tumors. To evaluate the proposed method, we have utilized two databases, namely, the Figshare and Brain MRI datasets, and attained an average accuracy of 98.8% and 98.5%, respectively. Both qualitative and quantitative analysis show that our approach is more proficient and consistent with detecting and classifying various types of brain tumors than other latest techniques.

## 1. Introduction

The brain tumor is a lethal disorder that causes mortality to many people every year [1,2]. A tumor is an irregular development of tissues inside the human skull that can disrupt the functionality of the nervous system and the human body [3]. According to the National Brain Tumor (NBT) Foundation research, in the USA, over 29,000 cases are identified with brain tumors, and approximately 13,000 victims die per annum [4]. Likewise, there are greater than 42,000 people who suffer from a brain tumor in the UK every year. Moreover, the statistics show that these patients vary in age, gender, health, and the tumor can occur somewhere in the brain [5].

With this substantial number of patients in the world, different medical imaging techniques such as magnetic resonance imaging (MRI), positron emission tomography (PET), computed tomography (CT), and X-ray are used for the diagnosis [6,7,8]. Among these MRIs is the ordinary non-intrusive imaging procedure widely adopted in the clinical routine because it does not use any damaging ionizing radiations [9,10]. Moreover, MRI provides images with high resolution and high contrast between soft tissues. MRI images are used for brain tumor diagnosis, surgical planning, and follow-up checkup to examine the growth of tumorous tissues. To analyze brain MRI images, usually, an expert radiologist manually identifies and segments the tumor region from MRI images based on visual inspection by employing anatomical and physiological knowledge [11,12]. This manual process is tedious, time-consuming, and prone to mistakes. Furthermore, it may experience delays due to the limited availability of radiologists. Early tumor detection and accurate segmentation are crucial, as they can increase the survival rate of patients and may save them from complex neurosurgical procedures [13,14].

With the advancements in the area of computer vision, there has been an increasing interest in computer-aided brain MRI analysis by researchers, radiologists, and clinical experts [15,16]. Brain tumor segmentation involves the extraction of the tumor region from healthy brain tissues. Various techniques have been proposed for the automated localization and segmentation of brain tumors. However, accurate and robust detection and segmentation of brain tumors is still a challenging task due to its complex anatomical structures, i.e., shape, size, and varying appearance from patient to patient [17]. Additionally, the tumor can appear anywhere in the brain, and its boundaries are often diffused with healthy brain tissues. Moreover, the presence of MRI artifacts such as noise and distortion added by imaging devices or acquisition protocols makes the accurate and automated delineation of the tumor region more difficult.

Numerous machine learning (ML) techniques have been proposed to execute automated brain tumor detection. These algorithms perform segmentation by classifying each pixel in an image as a tumor or healthy tissue built on the extracted key points. Thus, the accomplishment of these techniques is highly dependent on the extracted key points and classification approaches. MRI images have various features that are used for brain tumors segmentation purposes such as local histogram [18], image texture [19,20], and structure tensor eigenvalues [21]. The machine learning algorithms that include support vector machines [22], decision trees [23], principal component analysis [24], and conditional random forests (CRFs) [25] are applied for pattern identification in brain tumor segmentation.

Recent studies have successfully demonstrated the use of deep learning (DL) techniques with Convolutional Neural Networks (CNNs) for brain tumor segmentation [13]. These techniques can learn useful and more discriminative features automatically without the need for manual feature extraction and selection. A variety of CNN-based deep learning models such as 2D-CNNs [26] and 3D-CNNs [27] are proposed for tumor segmentation. The 3D-CNN models can better exploit 3D information from MRI as compared to 2D-CNNs; however, this is at the cost of network complexity and large memory requirement. Thus, 2D-CNNs have been commonly applied in the brain tumor segmentation approaches. The CNN-based approaches operate at the patch level, where the classification result of each patch is used to label its central voxel. These approaches handle each voxel label as independent of each other and thus lack the appearance and spatial consistency information. To consider the local dependencies of voxel labels, various techniques based on fully convolutional neural network (FCNN) [28] and cascaded CNN [29] architectures are proposed. Instead of predicting patch-wise probability distribution, FCNNs estimate pixel-wise probability distribution. FCNNs take complete images as input and apply segmentation in a single forward pass. In [29], cascaded CNN architecture calculates pixel-wise probability calculates and passes the attained results through CNN at early stages.

Accurate detection and classification of brain tumors is a challenging job due to extensive variations in the texture, size, and location of brain tumors. Furthermore, the light and color variations in the suspected samples further complicate the identification procedure. To deal with the challenges, we have proposed a custom CornerNet approach with DenseNet-41 as the base framework. Initially, the DenseNet-41 feature extraction module of improved CornerNet is employed to calculate the deep features. In the next step, the computed key points are localized and classified by the one-stage detector of CornerNet. We have evaluated the proposed solution over the two challenging datasets, namely, Figshare and Brain MRI, and confirmed through the reported results that our work is robust to brain tumor classification under the presence of size, color, texture, light, and brightness variations. Furthermore, improved CornerNet also exhibits effective classification accuracy under the occurrence of noise and blurring in the input images. Following are the main contribution of the proposed work:

I. Proposed an improved CornerNet approach with DenseNet-41 for keypoints extraction, which enhanced the brain tumor classification accuracy while reducing both the training and testing time complexity.
II. Precise detection of the cancerous region of the human brain because of the robustness of the CornerNet framework.
III. Proposed low-cost solution to brain tumor classification as CornerNet uses a one-stage object identification framework.
IV. Rigorous evaluation has been conducted in comparison to other state-of-the-art brain tumor detection approaches over standard databases comprising diverse images with several distortions, i.e., noise, blurriness, color, light changes, angle, size, and location variations, to demonstrate the efficacy of the presented approach.

## 2. Literature Review

This section presents a critical analysis of the existing latest brain tumor recognition and segmentation methods. Current brain tumor segmentation techniques related to the use of MRI images are categorized into two main types, namely, generative and discriminative models [30].

Generative models (GM) work by using previous knowledge related to the structure of both healthy and tumorous cells. In automated systems, for the unstructured tumorous shape, segmentation is a complicated task. GM identifies the tumor voxels as irregularity in the human brain. GM employs automated anatomical frameworks such as atlases, which were developed by constructing patterns through several healthy brains [31]. A better example of GM was presented by Prastawa et al. [32], in which an ICBM brain atlas had been evaluated against a given query sample. It worked by computing the posterior probabilities of three key regions of the human brain, namely, white and gray matter and cerebrospinal fluid. The computed features were compared to a predefined threshold to locate tumorous cells with the least posterior probability. Finally, a post-processing step was performed to maintain the special regularity [33]. To compute the probability of tumorous cells, many atlas-based approaches have been introduced in Khotanlou et al. [34] and Popuri et al. [35]. These methodologies had employed symmetry of the brain for their calculations. Some other researchers have implemented atlas registration together with tumor segmentation, as performing brain registering with significant malignance region to an atlas is a complicated job.

Now, discriminative models (DMs) have gained more focus for brain tumor segmentation [36]. Despite employing the past information, DM works by using the local information of a given input sample like pixel and neighborhood-based methods [21,37], texture-based keypoints [38], brain symmetry examination, the region of interest evaluations, and gradient computation in input samples. DM approaches are further categorized as machine learning (ML) or deep learning (DL) based techniques. In [39,40], a gradient computation-based approach has been employed for the detection and segmentation of brain tumors. The usage of mesh-free fractional partial differential equation enabled the techniques in [39,40] to select an arbitrary order of spatial derivative, which empowered them to identify the tumors of varying size and shapes; however, they may not perform well over large intensity changes within MRI images [39,40]. Hussain et al. [41] presented a framework for the automated segmentation of brain tumors. Initially, Anisotropic Filter (AF) [42] was applied over the input sample to eliminate the noise. After this, adjustment-based segmentation was used, which divided the areas of the tumor from the processed sample by utilizing a structuring element. Finally, the morphological operation was used to show the location of the tumor in an input image. The approach in [41] exhibits better tumor segmentation results; however, it is unable to identify a tumor of small size. Rajan et al. [43] presented a method for segmenting tumorous tissues from the human brain. After preprocessing, K-Mean clustering along with Fuzzy C-Means was applied over the input sample to obtain the image clusters. Then the co-occurrence matrix was used to compute the key points from obtained clusters. Finally, the SVM classifier was trained to detect the tumor region. The approach in [43] improves the computational complexity of the segmentation process; however, for large intensity variation in the input sample, it may not perform well. Sharif et al. [44] proposed a framework to detect and segment the brain tumor. The technique [44] consisted of four main parts. Firstly, preprocessing was accomplished to eliminate the noise in input samples. Secondly, the tumor part was segmented through an improved thresholding technique. Next, nine geometric and four Harlick key points were computed which were combined through a serial approach. In the fourth step, the Genetic Algorithm (GA) was applied for robust key points selection, which was employed to train the SVM classifier for locating the tumor. The approach in [44] exhibits better segmentation accuracy; however, it may not accurately detect the tumors along the boundaries of images. Kaya et al. [45] proposed a technique for segmenting cancerous tissues of the human brain. Initially, the five variants of principal component analysis (PCA) were applied over the input image for dimension reduction. In the second step, FCM and K-Means algorithms were applied over the processed images to segment the malignant region. The approach in [45] works well for tumor segmentation; however, it requires intensive training.

Recently, DL-based frameworks have been heavily explored for automated segmentation of brain tumors. Hu et al. [29] introduced a methodology for automated brain tumor segmentation. Initially, a multi-cascaded convolutional neural network (MCCNN) was employed to capture the local dependencies of labels. In the next step, conditional random fields (CRFs) were utilized to take the spatial contextual information and remove some false outputs for efficient segmentation. Finally, three segmentation frameworks utilizing the patches computed from various views, i.e., axial, coronal, and sagittal perspectives, were used to locate the brain tumor. The method in [29] is robust to brain tumor segmentation; however, it suffers from high computational cost. Iqbal et al. [46] proposed a technique to automatically segment brain tumors. Three DL-based frameworks, named CoveNet, a CNN architecture, LSTM, an RNN framework, and ensemble technique by combining both CoveNet and LSTM, were introduced. The approach in [46] is robust to tumor detection and segmentation; however, it is economically inefficient. Similarly, in [47], three DL frameworks named Interpolated Network, SkipNet, and SE-Net were applied for brain tumor segmentation. Qasem et al. [48] employed a watershed segmentation technique together with the KNN for brain tumor identification. The approach in [48] exhibits better detection accuracy over selected MRI samples; however, it is unable to precisely segment the tumor areas over the complicated images holding tumors with several organizational complexities. In [49,50], an encoder–decoder-based approach was presented to execute pixel-wise segmentation of tumorous cells from the healthy brain tissues. Similarly, in FR-MRINet [51], a 33-layer deep network together with an encoder and a fully connected decoder was introduced for tumor segmentation. Saba et al. [52] introduced an approach for automated segmentation of brain tumors by combining both hand-coded and deep features. Initially, the Grab cut approach was applied to segment the glioma using MRI. Then, local binary pattern (LBP) and histogram orientation gradient (HOG) was applied over the segmented samples to compute the hand-coded features, and for deep key points, VGG-19 was utilized. Later, both hand-coded and deep features were combined serially. Finally, based on computed key points, several classifiers, namely, SVM, KNN, logistic regression (LR), linear discriminant analysis (LDA), and decision tree (DT), were trained to classify the malignant and healthy brain tissues. The techniques in [51,52] show improved segmentation performance; however, this is at the expense of increased computational complexity. Thus, there exists a need to develop effective brain tumor detection methods that are robust to brain tumors having multiple structural complexities [53]. Table 1 present the comparison of the existing brain tumor detection and classification techniques.

## 3. Proposed Methodology

**Figure 1:**
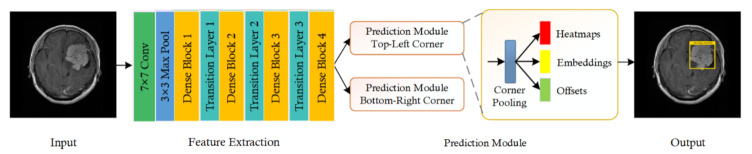
Proposed methodology diagram with four modules: Input, Feature Extraction using DenseNet-41 having four dense blocks and 3 transition layers, Prediction module to localize and classify the brain tumor by generating the bounding box along with confidence score, and finally, the obtained visual result as Output.

This section illustrates the proposed framework implemented for the identification of brain tumors. For a given input sample, the aim is to automatically recognize and detect the brain tumor without requiring any manual intervention. Our work is divided into two main steps: Firstly, we prepared the dataset by creating the annotations for input images to specify the exact location of tumors. Next, we trained the model using created annotations for tumor localization and classification. The proposed technique is based on a modified CornerNet model [54] that achieved state-of-the-art performance for object localization. We employed CornerNet with DenseNet-41 as its base network for feature calculation. During training, an input sample along with the bounding box annotation is passed to the improved CornerNet framework. The DenseNet computes the feature maps that are used by the CornerNet model to identify the class and location of the tumor. Finally, accuracies are estimated for all units as per metrics being employed in the field of computer vision. Figure 1 demonstrates the proposed methodology employed for tumor detection.

### 3.1. Annotations

**Figure 2:**
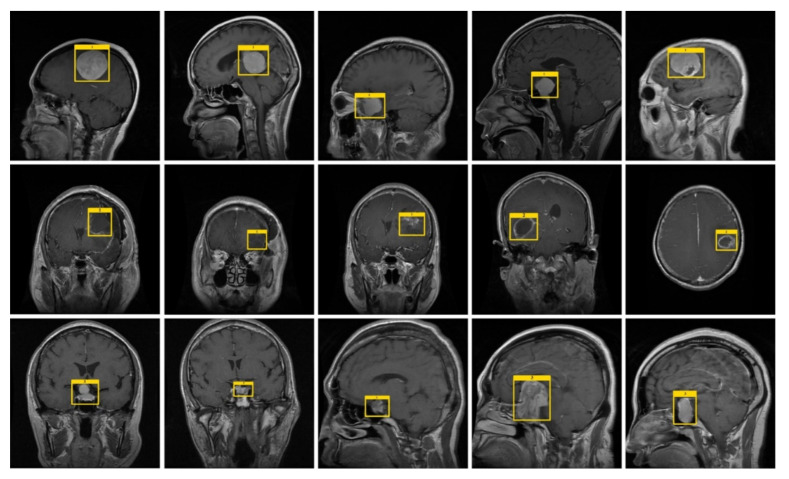
Sample annotated images.

To train the DL model, it is important to specify the exact location of tumors in input images. For this purpose, we have used the LabelImg tool [55] to create the annotations. Figure 2 shows a few samples of the annotated images. After the completion of the annotation process, an XML file containing the information about the tumor and their location coordinates in the images is obtained. Then, a training file is formed from the XML file, which is applied for the model training.

### 3.2. CornerNet Model

Our method is based on the CornerNet model [54] that is used for object detection. We have modified it for the detection of tumors to enhance model effectiveness and accomplish more accurate results. The CornerNet is a one-stage model that identifies the object bounding boxes by calculating corners, i.e., the Top-Left (TL) and Bottom-Right (BR) that is accurate and faster than other anchor-based approaches [56,57]. It uses a backbone network to calculate a group of feature maps that are manipulated to calculate two-channel (*C*), heatmaps, embeddings, and offset. The heatmap gives the likelihood of whether a specific location is a TL/BR corner of a certain class. The embeddings differentiate the keypoints pair and offsets for altering the location. Finally, the precise bounding boxes are acquired by picking the TL and BR points with the highest heatmap score, and pairing is executed based on belonged class and embedding distances.

The utilized CornerNet model detects the tumor using key points; thus, it eliminates the need of using a large number of anchor boxes for distinct object sizes that are commonly applied in other single-stage detectors such as SSD [58] and YOLO (v2, v3) [59]. Additionally, the aim of using the CornerNet model over two-stage detectors (RCNN [60], Fast RCNN [56], and Faster RCNN [57]) is that these methodologies use two distinct networks. Initially, a Region Proposal Network (RPN) is utilized for regions of interest (ROIs) creation, and then an independent network is employed for the classification of each ROI. These anchor-based methods necessitate high memory for calculation, which presents difficulty in hyperparameter choices such as the number, size, and aspect ratio of the anchors and thus results in decreased performance. The proposed CornerNet addresses the shortcoming of existing one-stage and two-stage detectors by engaging a corner calculation process that is computationally efficient.

### 3.3. Feature Extraction Using Customized Backbone Network

In this work, accurate and discriminative characteristics of the tumor are essential to differentiate them from the complex background and various variations in an image such as chrominance, intensity, contrast, illumination conditions, and blurring. The feature extractor of the CornerNet is the Hourglass104 network [54]. The drawback of this is that it is computationally complicated, i.e., it necessitates a vast number of parameters (187M parameters) and memory resources that unsurprisingly lead to low speed. To decrease the complexity, we adopt the DenseNet-41 [61,62] as the feature extractor to improve the extraction of features. The DenseNet-41 involves four densely correlated units with 41 layers and contains fewer parameters which provides it with a computational improvement than the Hourglass104. In DenseNet, all layers are precisely associated with each other, and feature maps from past layers are adopted to successive layers [63]. This model encourages feature reuse and develops the information stream all through the model, which becomes suitable to introduce complicated transformations efficiently for localization of tumor. The architectural description of DenseNet-41 is given in Table 2.

**Figure 3:**
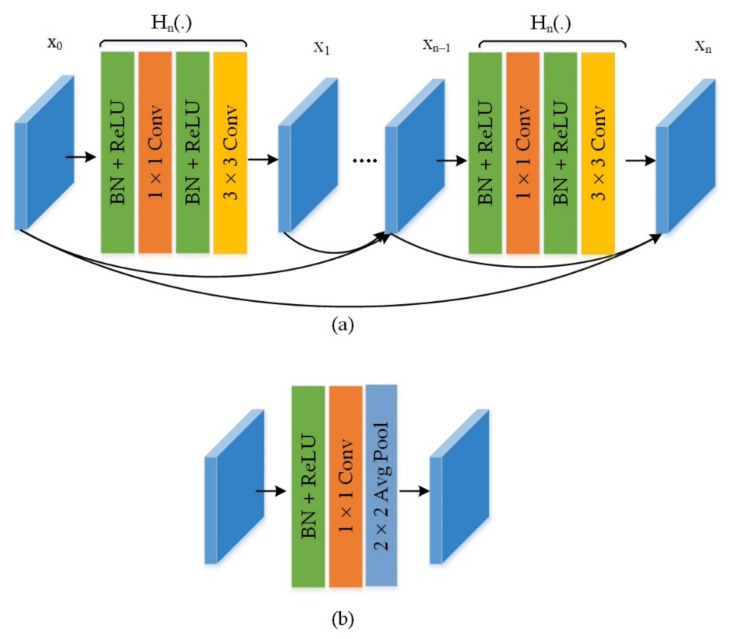
The structure of (**a**) Dense Block and (**b**) Transition Block.

The DenseNet encompasses multiple Convolutional Layer (ConvL), Dense Block (DB), and Transition Layer (TL). The DB is the key element of DenseNet appears in Figure 3. The z_0_ is the input layer encompassing f_0_ feature maps. The Hn(.) is a composite function involving three consecutive tasks, i.e., Batch Normalization (BN), a Rectified linear unit (ReLU), a 3 × 3 Conv kernel. Each H_n_(.) the procedure creates f feature maps, which are then spread to z_n_ succeeding layers. Since each layer gets all earlier layer feature maps as input, the input data of the n-th layer DB has f × (n − 1) + f_0_ feature maps. After the numerous dense links, the size of the feature map grows significantly. The TL is inserted between DB to decrease the feature dimension. It encompasses a BN and 1 × 1 ConvL followed by an average pooling layer, as displayed in Figure 3.

## 4. Results

### 4.1. Dataset

**Figure 4:**
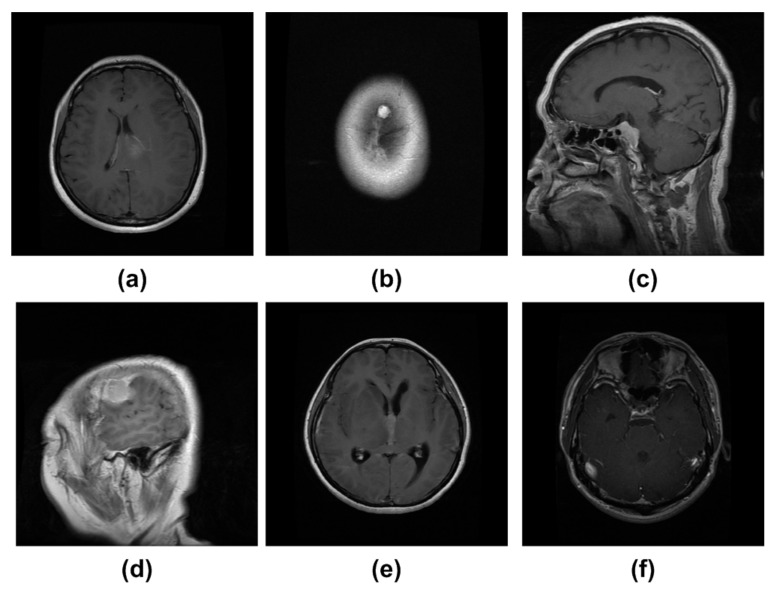
Sample dataset images. (**a**) Texture similarity; (**b**) noisy sample; (**c**) size variation; (**d**) blurry and brightness effect; (**e**) texture similarity; (**f**) low-contrast sample.

To assess the detection accuracy of our technique, we have employed two datasets, namely, the Figshare [64] and Brain MRI [65] dataset. The Figshare dataset is taken from [64] and is a larger size and challenging dataset for brain tumor identification. It comprises 3064 brain MRI images from 233 subjects of three types of, namely, Meningioma as “1”, Glioma as “2”, and Pituitary as “3”. More specifically, the Figshare database contains 930 images of class Pituitary, while there are 708 images are of Meningioma class, and the remaining 1426 samples are of Glioma tumor type. All images of this dataset have a matrix size of 512 × 512 pixels. The second used dataset, namely the Brain MRI database, is taken from [65] and has a relatively less number of samples. This dataset comprises a total of 231 MRI images with a matrix size of 845 × 845, in which there are a total of 155 tumorous samples. Both used databases are online available and contain images that are challenging in terms of textural complexity, color variations, noise, capturing devices, and bias field-effect, etc. Moreover, both datasets contain T1-weighted contrast-enhanced samples as the T1-weighted MRI images provide a bigger difference of the healthy and affected brain region. We have randomly split both datasets into 70–30%, where 70% of samples are used for training, while the remaining 30% of images are used for model testing. Figure 4 presents the sample images.

### 4.2. Evaluation Metrics

To assess the detection and classification accuracy of the presented framework, we employed several standard metrics, namely, intersection over Union (IOU), accuracy, precision, recall, and mean average precision (mAP). The accuracy of the proposed solution is calculated by using Equation (1):

(1) $$Accuracy=\frac{TP+TN}{TP+FP+TN+FN}$$

Equation (2) indicates the mAP formula, where *AP* shows the average precision of each class, and *t* is the query or test image. *T* represents the total test samples.

(2) $$mAP:=\sum_{i=1}^{T}AP(t_{i})/T$$

**Figure 5:**
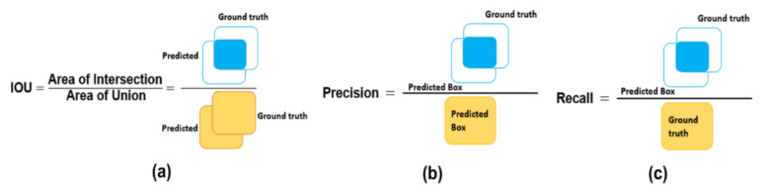
Geometrical representations (**a**) IOU, (**b**) precision, and (**c**) recall.

Figure 5 represents the pictorial form of IOU, precision, and recall.

### 4.3. Experimental Results and Discussion

**Figure 6:**
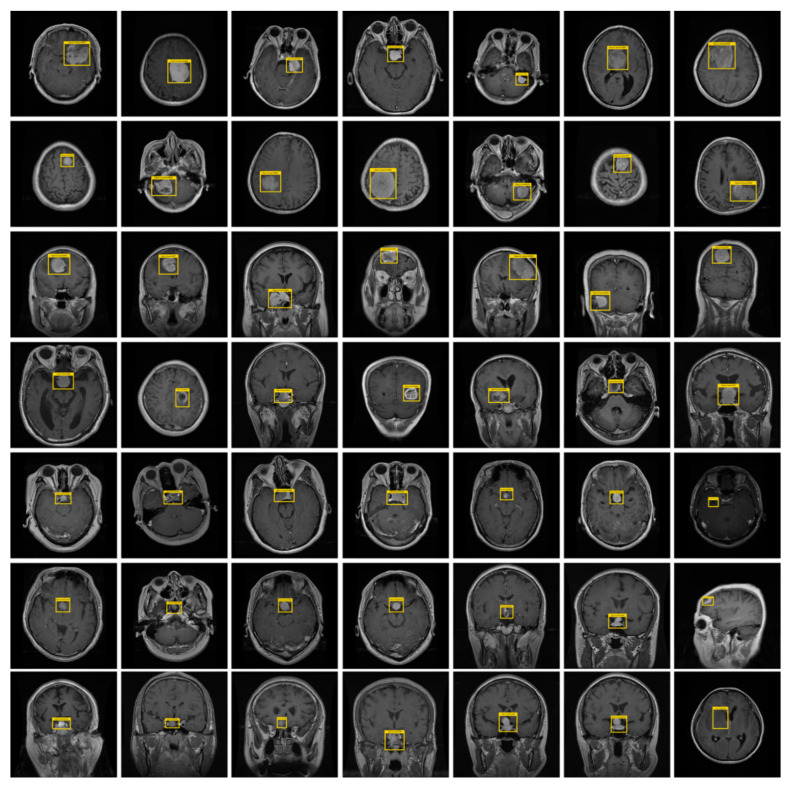
Samples images of localized tumor varying in size, color, location, and shape and under the occurrence of noise, blurring. and contrast variations.

Here, we have discussed the in-depth analysis of the acquired results. The precise recognition of numerous brain tumors is mandatory for designing an accurate computerized system for brain cancer cell detection. Therefore, we have evaluated the localization ability of the CornerNet framework by executing an experiment. We have performed the experiments over the samples of two datasets from [64,65]. The presented technique employed the CornerNet with DenseNet-41 at the feature extraction level, and obtained visual results are reported in Figure 6. It can be seen from the reported results that the presented CornerNet framework with DenseNet-41 backbone can accurately detect the tumorous region from the healthy part of the brain even under the presence of noise, blurring, and light variations. Furthermore, the improved CornerNet (with DenseNet-41) approach can accurately identify brain cancerous cells by dealing with the issues of varying position, structure, and sizes of tumors.

**Figure 7:**
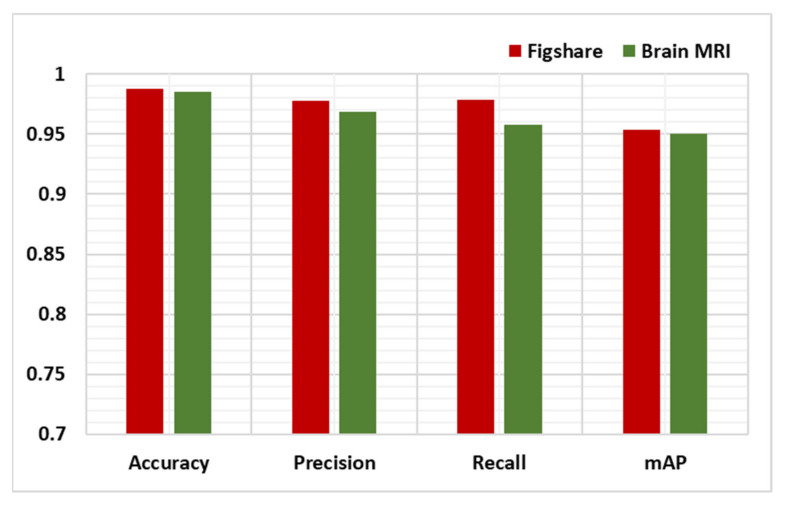
Proposed approach results over both datasets.

As discussed earlier, the detection power of the presented approach is analyzed by employing several performance metrics, i.e., precision, recall, accuracy, and mAP. To show the obtained values for our work over both databases, we have plotted the bar graph as shown in Figure 7. It can be visualized from Figure 7 that the presented method exhibits robust performance over the Figshare dataset in comparison to the Brain MRI database. More specifically, in the case of the Figshare dataset, we obtain an average accuracy value of 0.988, while in the case of the MRI dataset, we attain an average accuracy of 0.985 that is showing the robustness of our approach to brain tumor recognition. Moreover, we employed the mAP metric, which assists in determining that how much the system can recognize each type of brain tumor. In the case of the Figshare dataset, the system exhibits the mAP score of 0.953, while in the case of the MRI dataset, it obtains the mAP score of 0.950. It can be concluded from the employed qualitative and quantitative measures that the presented improved CornerNet can be reliably utilized for brain tumor detection and classification.

**Figure 8:**
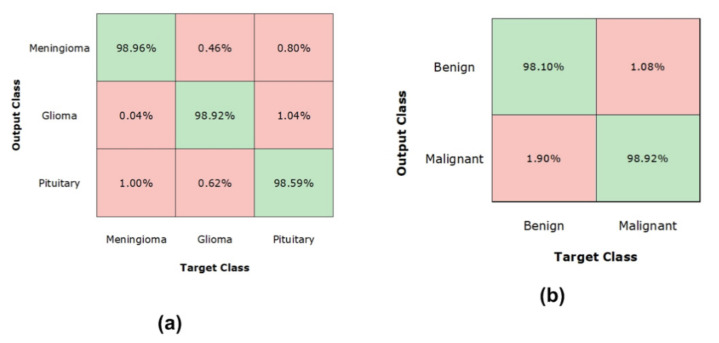
Confusion Matrix over (**a**) Figshare and (**b**) Brain MRI Dataset.

To further show the class-wise performance of the presented technique over both datasets, we have plotted the confusion matrix (Figure 8). The confusion matrix can better show the category-wise accuracy of the presented approach in terms of real and predicted class. In Figure 8, part (a) shows the class-wise TPR of the Figshare dataset, while part (b) shows the class-wise TPR for the MRI dataset. More specifically, the presented Custom CornerNet attains the TPR of 98.92%, 98.96%, and 98.59% for glioma, meningioma, and pituitary tumor types, respectively. Additionally, in the case of the Brain MRI database, the presented framework exhibits the TPR of 98.10% and 98.92% for the cancerous and non-cancerous brain cells, respectively.

### 4.4. Evaluation of DenseNet

We performed an analysis to demonstrate the significance of the presented framework’s dominance with other DL-based models used for brain tumor classification. To complete this task, we have compared the classification performance of our work with several base frameworks namely VGG-16 [66], VGG-19 [67], ResNet-50 [68], and DenseNet-121 [69], as explained in [70,71].

A comparison of the proposed solution with other DL-based frameworks in terms of model parameters, brain tumor classification accuracy, and execution time is shown in Table 3. It can be witnessed from the reported results that Custom CornerNet with DensNet is more robust to brain tumor recognition than the VGG-16, VGG-19, DenseNet-121, and ResNet-50 networks. Furthermore, Table 3 is clearly showing that the VGG-16 model has the highest number of framework parameters, while in terms of execution complexity, the DenseNet-121 is more expensive, while in comparison, the proposed approach with the DenseNet base model is economically robust as it takes only seconds for image processing. The major cause of the effective classification accuracy of the DeneNet is its shallower network that enables it to efficiently reutilize the network parameters without employing redundant key point maps. This configuration of the DenseNet model causes to minimization of the total number of parameters. While in contrast, the competitor approaches are suffering from the problem of high computational complexity and are not robust to several image post-processing attacks, i.e., light variations, color changes, noise, blurring, and varying tumor sizes. The proposed solution better address the limitation of existing approaches by introducing a more efficient keypoints extractor, namely DenseNet, which can better present the complex image transformations and can accurately deal with the sample post-processing attacks. Therefore, from the conducted analysis, it can be concluded that our framework shows better performance than the other DL-based methods, both in the form of tumor recognition accuracy and time complexity.

### 4.5. Comparison with Other Object Detection Methods

We performed an analysis to compare the brain tumor detection accuracy of the proposed method with other DL-based methods. To perform this task, we have taken both the one-stage and two-stage detection approaches for performance comparison. The two-stage methods require two steps for localizing and classifying the object of interest. Initially, these methods identify the location of brain tumors through creating numerous region proposals, which are narrow down in the next step, and then the resultant classification output is determined. While in the comparison, the single-stage approaches specify both location and an associated class of tumor at the same time.

We have compared the detection performance of our method with both two-stage (RCNN [72], Faster-RCNN [73], and Mask-RCNN [74]) and one-stage (YOLO [75] and SSD [58]) methods, and the results are reported in Table 4. The main issue of the RCNN approach is that it is suffering from high model training computational complexity, as it produces 2000 region proposals per suspected sample to perform the classification task. Moreover, RCNN lacks to have a learning process during the region proposal generation as it employs a hand-coded technique, namely selective search algorithm, which causes to produce several false candidate region proposals. The execution time for the RCNN model is about 0.47 s which is not acceptable for real-time object detection. The Faster-RCNN and Mask-RCNN have overcome the problem of false candidate region proposals generation of RCNN by introducing the automated region proposal network; however, due to their two-stage network, these approaches are computationally inefficient, while in the case of single-stage frameworks, the SSD and YOLO networks are unable to locate brain tumors of small sizes. The introduced framework better tackles the problems of both one-stage and two-stage approaches by presenting a Customize CornerNet with DenseNet as the backbone. The employment of DenseNet at the feature extraction layer of CornerNet enables it to compute the more discriminative set of image key points, which result in the efficient localization and classification of various categories of brain tumors. Furthermore, the one-stage detector nature of CornerNet has given it a computational advantage over other models as well.

### 4.6. Comparison with Other ML-Based Classifiers

To further explain the brain tumor classification performance of the proposed framework, we have designed another experiment to show a performance comparison with other ML-based classifiers. To accomplish this analysis, we have selected three well-recognized classifiers, namely, KELM [76], SVM [77], and GA [78], and the performance results are demonstrated in Table 5. From the table, the proposed custom CornerNet classifier acquired the best classification accuracy with a value of 98.7%, while the SVM classifier attained the second-highest accuracy value, 98%. At the same time, the KELM classifier showed a lower classification accuracy with a value of 93.68%. The comparison depicts that the introduced method is more robust to brain tumor detection and recognition as compared to other ML-based classifiers because of its power to better tackle the over-fitted training data.

### 4.7. Comparison with the State-of-the-Art Techniques

Here, we have compared the classification power of our approach with the other latest approaches, and the results in terms of accuracy values are reported in Table 6. To conduct a fair comparison, we have compared the average classification results of our approach with the average accuracy results of techniques given in [74,79,80,81].

Masood et al. [74] presented a custom Mask RCNN for the automated recognition of brain tumors and attained an average accuracy value of 98.34% along with the recall value of 95.3%. Similarly, Bodapati et al. [79] proposed a two-channel DL-based approach employing the InceptionResNetV2 and Xception networks for deep features computation, which is later classified into the tumor and non-tumor classes. The method [79] shows an average accuracy of 98.04. While the method in [80] used a DL-based approach, namely BrainMRNet, which employed the Otsu approach to determine the lobe region of the brain (i.e., left or right) containing a more concerted cancerous brain region. The method in [80] acquired an average tumor classification accuracy of 97.69% along with the precision and recall values of 96.24% and 96.22%, respectively. In [81], the author proposed a DL-based framework, namely Siamese neural network (SNN), for computing the deep features from the MRI images, which are later classified by the k-nearest neighbor (k-NN) attained the average accuracy and precision values of 92.6% and 95.3% respectively, while the presented framework acquired the average accuracy, precision, and recall values of 98.7%, 97.40%, and 96.9%, respectively, which are higher than all the comparative techniques. More specifically, the comparative approaches show an average accuracy value of 96.66%, while our method obtains an average accuracy value of 98.7%. Our approach gives a 2.03% performance gain in terms of classification accuracy. Moreover, in terms of precision and recall, our approach gives an average gain of 1.63% and 1.14%, respectively. Therefore, it can be concluded that the presented framework is more robust to brain tumor identification.

The reported performance analysis clearly shows that the proposed method outperforms the comparative approaches [74,79,80,81], as these techniques use very deep networks, which can easily encounter the problem of the model over-fitting, whereas the presented network employs DenseNet for computing the deep key points that extract more representative features and enables the Custom CornerNet to give a more accurate representation of brain cancerous regions over comparative approaches. Furthermore, the frameworks in [74,79,80,81] are economically inefficient than the presented approach; therefore, it can be summarized that the Custom CornerNet is more robust and effective to the brain tumor classification.

### 4.8. Discussion

Accurate and timely diagnosis of brain tumors cannot only save the patient’s life but also save them from complex and painful treatment procedures. In this work, we have introduced a DL-based framework, namely CornerNet with DenseNet-41 as the base network. We demonstrated the classification accuracy of our approach over the three types of brain tumors, namely Meningioma, Glioma, and Pituitary. We have evaluated the efficacy of our approach on two challenging datasets, namely the Figshare and Brain MRI datasets. We have randomly split both databases into training and testing sets with the ratio of 70% and 30% to assess their recognition power. The presented framework exhibits robust brain tumor detection and classification performance on both the Figshare and Brain MRI datasets. Both the qualitative and quantitative results confirm that our approach works well in comparison to the state-of-the-art approaches and is effective for tumors of varying sizes, angles, and locations. Furthermore, the work is efficient to recognize the brain tumors from the samples suffering from noise, blurring, and light and color variations.

In this work, we are focused on brain tumor classification; therefore, the approach produces results by drawing the bounding box around the ROIs. In the future, we will focus on designing such a framework that can draw the segmentation mask along with the classification results to clearly show the boundary between normal and tumorous brain tissues.

## 5. Conclusions

The proposed work presents a novel technique, namely improved CornerNet with DenseNet-41 as a backbone network for the automated identification and classification of brain tumors. More specifically, the DenseNet-41 is employed to compute the deep features from the suspected samples. In the next step, the extracted features are employed to train the CornerNet classifier to localize and recognize the various brain tumors. The proposed solution is capable of accurately differentiating the various classes of brain tumors. Furthermore, our method can easily deal with the challenges of varying size, position, and structure of brain cancerous cells. Moreover, the technique can identify the brain tumors under the presence of various post-processing attacks in the input images, i.e., noise, blurring, light, and intensity variations, etc. Experimental results clearly show that the custom CornerNet framework outperforms the existing state-of-the-art brain tumor classification approaches. In the future, we plan to test our model over other medical diseases and apply it to real-world scenarios and more challenging datasets to show its robustness.

## Figures and Tables

**Table 1:** Review of previous studies.

References	Method	Limitation
GM-based		
[32]	A posterior probability-based technique to identify the matching cases from history.	The work is not robust to the detection of varying shapes of brain tumors.
[34]	An atlas-based method was used to locate the presence of the brain tumor.	Needs the expertise of trained human experts.
[35]	An atlas-based approach together with brain symmetry was employed for detecting cancerous cells.	Performance degrades for samples having less texture information.
ML-based		
[39]	A gradient computation-based approach to localize the tumorous cells.	It may not perform well over large intensity changes within MRI images.
[41]	The AF method along with the adjustment-based segmentation technique was employed to identify the brain tumor.	Unable to detect a tumor of small size.
[43]	The K-Mean and Fuzzy C-Means clustering along with the co-occurrence matrix was employed for feature computation, while the SVM classifier was used for brain tumor classification.	The technique may not perform well over the samples with huge light changes.
[44]	The GA algorithm along with the SVM classifier was employed to detect the brain tumor from the MRI images.	This method may not accurately detect the tumors along the boundaries of images.
[45]	The PCA technique along with FCM and K-means clustering was used for locating the cancerous tissues of the human brain.	The approach requires huge training data.
DL-based		
[29]	The MCCNN framework along with the CRFs was used for brain tumor detection.	The method is economically inefficient.
[46]	An approach merging both combining both the CoveNet and the LSTM framework was introduced to identify the brain regions containing the tumor.	The approach is suffering from high computational complexity.
[47]	Three DL frameworks named Interpolated Network, SkipNet, and SE-Net were applied for brain tumor segmentation.	The work may not be generalized well to real-world scenarios.
[19]	A watershed segmentation technique together with the KNN for brain tumor detection.	This method is not robust to identify the brain tumor from the MRI samples having organizational complexities.
[50]	An encoder–decoder-based method was used for identifying the tumorous cells.	The technique is not robust to brain tumors of small sizes.
[51]	A 33-layer deep network was used to locate the cancerous brain cells.	The technique is computationally expensive.
[52]	The LBP, HOG descriptors along the VGG-19 framework were used for feature computation. While the SVM, KNN, LDA, LD, and DT were used for classification.	The approach may not perform well over the samples with extensive color changes.

**Table 2:** Architecture of DenseNet-41.

Layer		Operator	Stride
Convolutional Layer		7×7 conv	2
Pooling		3×3 avg_pool	2
DB1		$\left(\begin{array}{c} 1\times 1\;\mathrm{conv}\\ 3\times 3\;\mathrm{conv} \end{array}\;\right)\times 3$	1
TL1	Convolutional Layer	1×1 conv	
	Pooling Layer	2×2 avg_pool	
DB2		$\left(\begin{array}{c} 1\times 1\;\mathrm{conv}\\ 3\times 3\;\mathrm{conv} \end{array}\;\right)\times 6$	1
TL2	Convolutional Layer	1×1 conv	
	Pooling Layer	2×2 avg_pool	
DB3		$\left(\begin{array}{c} 1\times 1\;\mathrm{conv}\\ 3\times 3\;\mathrm{conv} \end{array}\;\right)\times 6$	1
TL3	Convolutional Layer	1×1 conv	
	Pooling Layer	2×2 avg_pool	
DB4		$\left(\begin{array}{c} 1\times 1\;\mathrm{conv}\\ 3\times 3\;\mathrm{conv} \end{array}\;\right)\times 3$	1
Classification Layer		7×7 avg_pool FC layer	

**Table 3:** Comparison with base models.

Model	No of Parameters (Million)	Accuracy (%)	Execution Time (s)
VGG16	119.6	98.06	1051
VGG19	143.6	97.97	1312
ResNet50	23.6	96.67	1583
DenseNet121	7.1	98.15	2165
Proposed	6.1	98.7	1022

**Table 4:** Comparative analysis with other techniques.

Method	Evaluation Parameters			
	Accuracy	mAP	Sensitivity	Time (s)
**Two-Stage Frameworks**				
RCNN	0.920	0.910	0.950	0.47
Faster RCNN	0.940	0.940	0.940	0.25
Mask-RCNN	0.983	0.949	0.953	0.20
**One-Stage Frameworks**				
YOLO	0.873	0.830	0.808	0.25
SSD	0.893	0.851	0.824	0.23
Proposed	0.987	0.952	0.969	0.19

**Table 5:** Comparison with ML-based methods.

Classifier	Accuracy (%)
Deep features + KELM [76]	93.68
Deep features + SVM [77]	98.00
Deep features + GA [78]	94.20
Proposed	98.70

**Table 6:** Comparison with state-of-the-art methods.

References	Method	Accuracy (%)	Precision (%)	Recall (%)
[74]	Custom Mask-RCNN	98.34	-	95.3
[79]	Two-Channel DNN	98.04	-	-
[80]	Attention module, Hyper-column technique, and Residual block	97.69	96.24	96.22
[81]	SNN + KNN	92.6	95.3	-
Proposed	CornerNet with DenseNet-41	98.7	97.4	96.9

## Data Availability

Not applicable.
